# The role of collaborative learning in resilience in healthcare—a thematic qualitative meta-synthesis of resilience narratives

**DOI:** 10.1186/s12913-022-08451-y

**Published:** 2022-08-26

**Authors:** Cecilie Haraldseid-Driftland, Stephen Billett, Veslemøy Guise, Lene Schibevaag, Janne Gro Alsvik, Birte Fagerdal, Hilda Bø Lyng, Siri Wiig

**Affiliations:** 1 SHARE - Centre for Resilience in Healthcare, Faculty of Health Sciences, University of Stavanger, N-4036 Stavanger, Norway; 2 School of Education and Professional Studies, Griffith University, Mount Gravatt, QLD 4122 Australia

**Keywords:** Resilience, Healthcare, Quality, Collaborative learning, Organizational learning, Systems perspective

## Abstract

**Background:**

To provide high quality services in increasingly complex, constantly changing circumstances, healthcare organizations worldwide need a high level of resilience, to adapt and respond to challenges and changes at all system levels. For healthcare organizations to strengthen their resilience, a significant level of continuous learning is required. Given the interdependence required amongst healthcare professionals and stakeholders when providing healthcare, this learning needs to be collaborative, as a prerequisite to operationalizing resilience in healthcare. As particular elements of collaborative working, and learning are likely to promote resilience, there is a need to explore the underlying collaborative learning mechanisms and how and why collaborations occur during adaptations and responses. The aim of this study is to describe collaborative learning processes in relation to resilient healthcare based on an investigation of narratives developed from studies representing diverse healthcare contexts and levels.

**Methods:**

The method used to develop understanding of collaborative learning across diverse healthcare contexts and levels was to first conduct a narrative inquiry of a comprehensive dataset of published health services research studies. This resulted in 14 narratives (70 pages), synthesised from a total of 40 published articles and 6 PhD synopses. The narratives where then analysed using a thematic meta-synthesis approach.

**Results:**

The results show that, across levels and contexts, healthcare professionals collaborate to respond and adapt to change, maintain processes and functions, and improve quality and safety. This collaboration comprises activities and interactions such as exchanging information, coordinating, negotiating, and aligning needs and developing buffers. The learning activities embedded in these collaborations are both activities of daily work, such as discussions, prioritizing and delegation of tasks, and intentional educational activities such as seminars or simulation activities.

**Conclusions:**

Based on these findings, we propose that the enactment of resilience in healthcare is dependent on these collaborations and learning processes, across different levels and contexts. A systems perspective of resilience demands collaboration and learning within and across all system levels. Creating space for reflection and awareness through activities of everyday work, could support individual, team and organizational learning.

**Supplementary Information:**

The online version contains supplementary material available at 10.1186/s12913-022-08451-y.

## Background

Healthcare services worldwide are provided in increasingly complex, and always changing circumstances [1]. These challenges and changes comprise those associated with i) the healthcare issues faced by the population, ii) the dynamic character, conditions, and existing health of that population, iii) emerging therapies and practices, iv) shifts in policies and practices associated with the physical, organisational, environmental, and v) social circumstances in which healthcare provisions are enacted. Addressing such complex changing circumstances, in terms of patients’ needs, emerging therapies and practices and settings, therefore, becomes a necessity for all healthcare systems and healthcare professionals [2]. Recently, these complexities and constant changes have become more frequent and are of greater amplitude, such as illustrated by the Covid-19 pandemic. This implies the ability to enact adaptions and address these changes while continuing high quality service provision [3–6]. Resilience in healthcare is defined as the capacity to adapt to challenges and changes at different system levels, to maintain high quality care [7]. For healthcare organizations to strengthen their ability for resilient performance, a significant level of continuous learning is required [7, 8]. Lately, there has been a growing consensus amongst safety experts, system engineers and healthcare professionals, calling for a new approach to learning, which is not just focused on learning from what goes wrong in healthcare, but also to take a more proactive and reflective approach through learning from what goes right in ordinary work processes [9–12].

Resilience in healthcare builds on theory from other sectors such as societal safety, engineering (resilience engineering), social ecology and psychology, and is a theoretical perspective that explores how complex adaptive systems cope, respond, and adapt to stress [5, 7]. Different from more traditional ways of studying and explaining healthcare quality, resilience in healthcare tends to focus on successful outcomes rather than failures [13, 14]. This approach provides a more holistic and dynamic understanding of healthcare systems as it attempts to understand and explain the underlying processes of what contributes to the ability to handle unforeseen events, changes, and innovations. Recent studies report that capacities such as coordination, involvement, communication, leadership, and learning are key capacities for resilience in healthcare, all of which build on the need for engaging and interacting with a range of different stakeholders at different system levels in a collaborative effort [15]. This is not surprising given that the complex nature of healthcare organizations means that healthcare provision has increasingly become a shared effort amongst the different stakeholders who work collaboratively, often across different professions, levels and contexts to address patients’ and families’ needs [16]. More specifically, the ability to adapt and respond to challenges and changes relates to the ability to both work and learn, collaboratively, which enables healthcare professionals to actively develop a shared understanding and provide quality care [15].

This high level of interdependence amongst healthcare professionals and other healthcare stakeholders mandates that enhanced collaborative learning skills, such as good communication and coordination of work tasks, both within and across different professions, teams, and team members can play key roles in improved healthcare performance [17, 18]. Improved healthcare provision is, therefore, not just about learning as individuals, but also about working and learning collaboratively across stakeholders and system levels. There is no single definition of collaborative learning through work. However, there is consensus that it comprises a group of learners, working together to solve a problem or complete a task [19] and it is through these activities and interactions that participants’ learning arises. In particular, it is through this joint problem solving, between more and less experienced interlocutors, that new insights, procedures, and sentiments are made accessible and learnt. Moreover, these engagements are both generative of new knowledge and extend what learners know, can do and value [20, 21]. This recognition led to education models such as Reciprocal Teaching and Learning [22], Cognitive Apprenticeships [23] and Guided Learning at Work [24].

In a collaborative learning setting, the learners are both informed by and challenged through listening to different perspectives, defending own ideas and creating own unique understandings, based on their experiences. Learning, therefore, occurs continuously in healthcare systems through healthcare professionals engagement in clinical work, and by interacting with co-workers, patients, and other stakeholders [25]. Collaborative learning through engaging reciprocally with others through work practices such as teamwork and problem solving is also central in quality processes [26, 27] as it is often in response to novel challenges of emerging problems. Researchers have, therefore, suggested practitioners’ on-going learning across their working lives, and in particular collaborative learning, as a prerequisite to operationalize resilience [5, 6, 15, 28, 29].

Previous research shows how adaptation is linked to learning within the field of resilience [8, 30–32]. Yet only limited systematic attention has been given to the collaborative learning element in these adaptive capacities [5, 7]. Most frequently, resilience studies adress learning as an outcome, pointing at specific adaptive practices to handle capacity-demand misalignments, such as workarounds [33], secret second handovers [34], or next of kin agency [35, 36]. More recently, some studies have focused on developing specific tools for strengthening resilient capacities, such as serious games [37–40] and reflective spaces or narratives [12, 28]. However, resilience studies are frequently tightly focused on individual learning,- such as people-technology interaction [41], or openness for change,- [42], with only limited focus on team learning or collaborative learning approaches. Furthermore, only a few studies focus on strengthening resilient capacities in a team setting [43–45]. Recent studies have, therefore, indicated that to advance the field of resilience in healthcare there is a need to develop collaborative learning tools that aid healthcare organizations in strengthening their resilience performance through collaborative efforts that helps create awareness of what goes right, and understanding the underlying factors contributing to the desired outcome [15, 46, 47].

Given the potential of these collaborative learning elements to promote resilience there is a need to explore the underlying collaborative learning processes and how and why collaborations occur during adaptations, trade-offs, and improvisations as a response to disruptions, challenges, and changes [7, 46]. Exploring these underlying processes is the key to understanding how learning resources should be developed to translate resilience into practice and strengthen resilience capacities [46], and our study addresses this knowledge gap.

### Aim and research questions

The aim of this paper is to describe and discuss collaborative learning processes in relation to resilient healthcare based on investigation of empirical findings from diverse healthcare contexts and levels.

The research questions are:

1. For which purposes do stakeholders in the healthcare system collaborate?
2. Which activities and interactions constitutes those collaborations?
3. Through which processes does collaborative learning arise?

This article contributes knowledge on the identification of how healthcare professionals and other stakeholders in the healthcare system collaborate and interact when responding and adapting to challenges and changes. This contribution is advanced from a resilient healthcare perspective to elaborate on the role of collaborative learning.

## Methods

### Design, sample selection and data collection

This study is an element of the longitudinal research programme: Resilience in Healthcare (RiH), (2018–2023) [5]. The overall aim of the research programme is to apply a collaborative interactive research design to establish a comprehensive RIH framework aimed at identifying and strengthening resilience in healthcare. The collaborative interactive design will, through iterative cycles of different research activities (i.e. workshops, focus group interviews, individual interviews), bring together key actors (i.e. multidisciplinary researchers, practitioners, technology designers) in multiple phases of development, implementation, evaluation and improvement [5]. In this article, we report on an explorative study of 14 empirical research projects from diverse healthcare settings, undertaken as part of the first explorative phase of the overall research programme [5]. Please see supplementary file 1 for details of the empirical research projects included.

During the explorative phase, the aim was to move beyond single-site, case-based studies to review resilient capacities and collaborative learning processes across different healthcare contexts and levels. The process therefore commenced with the screening of a sample of 50 research projects (i.e., post doctor projects, PhD projects and research project). The research projects were all associated with the SHARE-Centre for Resilience in Healthcare, in Norway. SHARE is Norway’s leading research Centre within resilience, quality, and patient safety. The Centre has studied a vast range of different resilience and patient safety related issues over the last 15 years, within a range of healthcare settings and focused on a variety of different stakeholders. This variety of projects, contexts and stakeholders, therefore, represents an opportunity to explore collaborative learning across levels and contexts, thereby advancing the research beyond single-site, case-based studies towards cross-context multisite studies as suggested in the literature [48]. The 50 projects were comprised of former and ongoing granted research projects at both micro, meso and macro level, stemming from a variety of healthcare settings such as homecare, nursing homes, hospital, education, and prehospital care. Furthermore, projects include multiple stakeholders (i.e., patients, next of kin, manager, healthcare professionals, students, and regulators) and a variety of quality dimensions (i.e., patent safety, clinical effectiveness, coordination, patient centredness, patient safety). While all projects are related to healthcare and patient safety, it varied to which degree they focused on quality and resilience. Before being subjected to further analysis all projects were therefore reviewed for relevance for the overall RiH-project through a screening process using an established screening protocol [5].

The screening process entailed a six-step process with the aim of considering each project’s relevance for further inclusion in relation to quality and resilience. The six steps consisted of:

1. Determine all projects with a SHARE affiliation
2. List all projects for initial screening
3. Initial screening according to the Quality and Resilience Trigger Tool
4. Second level screening of projects
5. Group consensus for final inclusion
6. Summary of final project inclusion

In step 3, Quality dimensions refers to patient experiences, patient safety, clinical effectiveness, and care coordination, while Resilience dimensions refers to individual-, team/unity-, organizational- or larger system capacity, that contributes to the capacity to adapt to challenges and changes at different system levels, to maintain high quality care [7]. For further details of the Screening Protocol and a Quality and Resilience Trigger Tool Please see Aase et al. [5] data supplement one and two. Based on this screening process, 14 projects were developed into narratives and included in the study.

The 14 projects have generated a total of 40 published articles and 6 PhD synopses. The data were collected from journal websites, databases, and a publicly available database for Norwegian PhD theses, between February 2020 and September 2020. For details about the selected projects, please see additional file 1.

Based on a predefined template developed by the research team, a narrative was prepared for each of the 14 research projects The template that dictated how the narrative was to be written entailed (1) defining the phenomenon of resilience, (2) describing setting, system level, stakeholders involved, professions, competence levels and contextual conditions surrounding the project, (3) Describing the content of the project in 4–7 pages. Defining the phenomena of resilience was done according to C Macrae and S Wiig [49] four dimensions of resilience:

- Resilience for what? (What goals and objectives is resilience supporting?)
- Resilience to what? (What triggers, activates, or necessitates resilience?)
- Resilience of what? (What materials and resources underpin resilience?)
- Resilience through what? (What mechanisms, activities and interactions enact resilience?)

All narratives were developed by pairs of researchers and subjected to an iterative process of discussions and refinement to validate whether all important aspects of the project were included. The 14 narratives resulted in a total of 70 pages of text. (Please see [50] p. 4–5 for more details).

### Analysis

Analysis of the 14 narratives was undertaken based on a thematic meta-synthesis approach inspired by J Thomas and A Harden [51]. The analysis process is performed in three stages, Stage one; coding text, Stage two; developing descriptive themes and Stage three: generating analytical themes. The analysis process was guided by the research questions and each of the 14 narratives were analysed to identify: i) purposes of the collaboration amongst involved healthcare professionals at different levels (i.e., *why* they collaborated), ii) which activities and interactions constituted those collaborations (i.e., *how* they collaborated), and the specific processes through which learning arises (i.e., which activities they could potentially learn from). In Stage One all authors read the entire data material, while author CHD and SB coded the narratives and grouped the codes into suggestions for preliminary themes, separately. During this stage the authors extracted each segment of the narratives that they found relevant for the purpose of the study and classified them into three different themes related to the three research questions (purpose, how and why). All segments were given a code. CHD then checked for consistency of interpretation throughout the codes. In the second stage there was a need for development of new themes since several of the codes did not fit into the preliminary themes, while some themes contained a lot of codes, while other themes covered few codes. All codes under each theme were then regrouped by CHD into six new themes, and new codes were generated when needed. In step three all authors discussed new themes and subthemes in two joint workshops. After each workshop CHD regrouped the codes under each new theme and subthemes. Finally, all authors agreed upon three main themes and eight subthemes to best represent the content of the data. In Table 1, the three stages are set out in the left column, a description of what they comprised in the middle column, and participants in the analysis in the right column. The findings from this three-staged process comprised the identification of themes that permitted the categorization and analysis of the data.

**Table 1:** Description of stages of the thematic meta-synthesis process

Stage	Description of analysis process	Participants
**1. Coding text**	- Inductive, line-by-line coding to capture the meaning and content, keeping the synthesis close to the original text. 138 codes by CHD and 146 codes by SB -Grouping codes into suggestions for preliminary themes (three themes)	CHD and SB separately
	-Check for consistency and interpretation throughout the codes	CHD
**2. Developing descriptive themes**	- Re-grouping codes into new themes (six themes) -Generating new codes when needed Drafting summary of findings	CHD
**3. Generating analytical themes**	- Author discussions, comparing and revising themes (six themes became three themes with eight sub themes)	All authors
	-Reorganizing codes under new themes	CHD
	**-** Theme labels were refined and revised, making sure they reflected the content	All authors

## Results

The findings are presented through aligning them with the research questions addressing: 1) purposes for collaboration, 2) activities and interactions that constitutes the collaborations and 3) processes through which learning arise. An overview of the findings is provided in Table 2, as presented under two columns, the right-hand one presenting the themes and the column to its left setting out associated sub themes.

**Table 2:** Overview of themes and subthemes

Themes	Subthemes
**Purposes for collaboration**	Respond and adapt to change
	Maintain processes and functions
	Improve service quality and patient safety
**Collaborative activities and interactions**	Exchange information
	Coordination, negotiation and aligning needs
	Develop buffers
**Processes in which collaborative learning arise**	Activities of everyday work
	Intentional educational activities

### Purposes for collaboration

The three main purposes for stakeholders to collaborate are: i) responding and adapting to change, ii) maintain processes and functions, and to iii) improve service quality and patient safety. These are now presented in turn.

#### Respond and adapt to change

The most frequent purpose of collaboration is responding and adapting to change, due to the constant change of context that all the different stakeholders, both within and across system levels experience.

Some changes result in collaborative efforts both within and across all levels and settings, such as changes in legislation, government-initiated reforms (in Narratives 3 and 4), budget cuts, introduction of new tools (in Narratives 5 and 12), or simulation and training (in Narratives 5 and 7). Other changes result in collaborative efforts only in one level such as within a team or a specific context (in Narratives 1–10). Examples of such changes are caring for a patient with deteriorating health; involving next of kin in the care process; new leadership, alternations in team composition, or handling peak activity situations or excess workload. The adaptations take different forms such as reallocation of responsibility or resources (in Narratives 1,7,9), or local adaptations in procedures due to perceived flaws, insufficiencies, or inability to adhere to original outlines (in Narratives 13,7,11,12).

Responding and adapting to change is a task that demands interactions and collaboration between different stakeholders, across settings, between individuals and groups, within and between groups, or between individuals and systems, equipment, technology, or context. These changes are not, and cannot be addressed by individuals alone, as they require interaction and shared activities with others.

#### Maintain processes and functions

Next to responding and adapting to change, the stakeholders’ collaborative activities often have the purpose of maintaining normal functions and processes (in Narrative 1–14). This requirement generates a high degree of daily collaborations that are a product of how work is organized. All systems levels, from macro, to meso, to micro are involved and are interdependent on each other’s collaboration to perform everyday activities. For example, admissions and discharges are dependent on team contributions from stakeholders, such as physicians, nurses, next of kin and the patient themselves, but also on collaborations between stakeholders across different contexts and levels, such as leaders and healthcare professionals, home care providers and hospitals (in Narrative 3,4). Collaboration is needed because a high degree of the tasks is dependent on those different stakeholders to inhabit different skills, knowledge, and responsibilities. For example, a team of anaesthetists, operating nurses and surgeons is needed to perform a surgery (in Narrative 6). Regulatory bodies, similarly, depend on collaborations with hospital departments, management, and healthcare professionals to ensure adherence to policies and guidelines (in Narratives 12,14). So, in these ways, standard and enduring clinical practices are dependent upon collaborative working and learning.

#### Improve service quality and patient safety

The third purpose for collaboration is when collaborations intentionally seek to improve service quality and patient safety. These interactions relate not only to a necessary adaptation to a change or the maintenance of a process or function but aim at making an intentional and specific effort to improve the quality and safety of processes or functions.

Collaborative efforts to improve quality and safety include introducing activities aimed at reducing variability and flexibility in clinical practice. Specific examples here include fixed work lists, routines, procedures, or triaging, to minimize variability and, thereby assist in ensuring better work practices, all of which highly involves a collaborative element. For example, provision of digital access to national guidelines to increase the chance of adherence (in Narrative 12). Collaborative improvement efforts also include structures designed to reduce potential risk such as the development of risk-based selection criteria in deciding where women in labour give births (in Narrative 1). Improvement efforts also involved prioritizing support and development opportunities (in Narratives 1,5,12), and the provision of meeting places for knowledge exchange, aiming at improvement of the healthcare services provided (in Narratives 4,7,8).

Adapting, maintaining, or improving services are not mutually exclusive. In fact, they are often intertwined, and shift rapidly. Stakeholders can collaborate for one, several or all purposes at once. For example, after the introduction of a change such as a peak activity situation, stakeholders could both adapt to the change in demand through reallocating resources, to maintain an adequate level of healthcare service quality, while at the same time changing team composition to improve collaborations and improve healthcare provision (in Narrative 1). In these ways, improving care quality and safety inherently aligned with collaborative working and learning.

### Collaborative activities and interactions

To achieve or work towards the desired purposes of a collaboration, the participants need to make use of and optimise a range of different activities and interactions. These have been divided into three different categories; 1) exchange information, 2) coordinate, negotiate and align different needs, and 3) develop buffers, which are now presented in turn.

#### Exchange information

Information exchange is found to be the most extensively used type of collaborative activity. The stakeholders collaborate about information exchange within disciplines, between disciplines and across multiple settings and levels. The information exchange is manifested in different ways, ranging from teaching next of kin to observe specific changes in the patient’s condition (in Narrative 7), to meetings with municipal managers to discuss major reforms such as change of care district (in Narrative 2).

There are different, often multiple goals for the information exchange, such as securing safe knowledge transfer (in Narrative 9) or preventing adverse events (in Narrative 7). However, most often the goal of the information exchange is to optimize or improve healthcare services (in Narratives 5, 8, 7, 2,13).

Participants make use of both explicit and tacit information exchange practices to interact with patients, clinicians, next of kin, and technology to be able to optimise care, anticipate, prepare, and plan for ongoing and future events.

#### Coordination, negotiation and aligning needs

In every collaboration the involved parties hold different needs, preferences, and desired outcomes of the collaboration. A large part of the collaborative efforts, therefore, is coordinating, negotiating, and aligning the different needs within each collaboration. This means that a lot of their activity concerns verbal and non-verbal interactions both within and between system levels and contexts to clarify trade-off situations, prioritize and make decisions. For instance, this is seen when regulatory investigators involve next of kin in investigations of adverse events, with the dual purpose of extracting new information, acknowledging the importance of involving different stakeholders and introducing a quality assurance element in the process (in Narrative 8).

Lack of resources often fosters negotiation amongst different stakeholders concerning which tasks to prioritize or whether to provide poorer care or involve next of kin in care activities to compensate for lack of healthcare professionals (in Narrative 7), which service levels have the responsibility for what (in Narratives 3,4,14), or which perspectives and needs that should be prioritized (in Narratives 1, 2, 3, 4, 9, 7, 11,12). An example of negotiations relates to granting patients an x-ray that the physician believes is unwarranted, thus using precious resources, yet providing the patient with the sense of being heard and involved (narrative 12).

Different stakeholders are placed in numerous situations where they need to collaborate, to align different needs and handle trade-off situations and carefully navigate through different ethical and practical dilemmas such as who to involve, when and why, and who have responsibilities for what. In these ways, collaborative working, and learning are essential for realising these kinds of needs that are central to care quality and safety.

#### Develop buffers

Collaborative activities also concern the creation of buffers in the system, aimed at anticipating future events and thereby, proactively preventing problems or aiding healthcare provision. These buffers are developed at different levels and involve outcomes in diverse forms and shapes and are either part of organizational structures or dependent on individual efforts. Organizational structures comprise scheduled simulation-based scenario training, (in Narrative 5) to be better prepared for diverse situations or having a designated section coordinator who can handle peak activity situations through accessing and reallocating resources (in Narrative 1). Individual efforts are often dependent on facilitators and individuals’ competencies, who collaborate with their surroundings to create local buffers such as combining experienced and inexperienced staff to make more robust team compositions (in Narrative 1.6). So, these anticipatory and proactive processes are reliant upon collective and collaborative expertise within the healthcare setting, again underpinning the importance of these forms of interdependent working and learning.

### Collaborative processes in which learning arise

The stakeholders involved are constantly engaged in a complex network of collaborative activities. These activities are divided into i) activities of everyday work, and ii) Intentional educational activities.

#### Activities of everyday work

Across settings and levels, the stakeholders engage in a range of collaborative practices as a result of the activities of their everyday work. These comprise activities such as debriefs, information sharing/exchange, consultations, discussions, prioritizing, anticipation, responding, and clarification of needs. These interactions occur both among different practitioners (e.g., doctors, radiologists, specialized nurses, regulators, or leaders), between different stakeholder groups (e.g., practitioners, patients and next of kin), between diverse types of stakeholders from the same group (e.g., managers, patients and next of kin), and between and within organizations across different levels of the healthcare system (in Narratives 1–14).

The common denominator of all these activities is that they entail interactions or activities where knowledge is expressed, shown, or shared and thus provides learning opportunities for the ones involved. Examples of collaborative activities of everyday work include when next of kin provides information about a patient’s status to the nurse that comes on duty (in Narrative 6); or a discussion amongst nurses regarding dosage and administration of a drug (in Narrative 8). So, again, these practices require interaction and interdependence amongst participants.

#### Intentional educational activities

Intentional educational activities include the types of learning activities that are planned or scheduled with an intention to educate the involved parties. Although present in the data material, this type of learning activity is less evident in the findings compared to learning as a result of everyday work activities.

Examples of intentional educational activities are workshops, debriefs, seminars or simulation-based training exercises. The purpose of these activities mainly relates to quality and safety improvement efforts and is limited to one level and setting such as simulation-based activities at a ward or department with the intent to practice and improve specific skills (in Narrative 1,5). However, there is also evidence of intentional educational activities which gather stakeholders such as leaders and staff, across levels and across settings such as hospitals, government and community care (in Narratives 2,4,8).

## Discussion

**Fig. 1:**
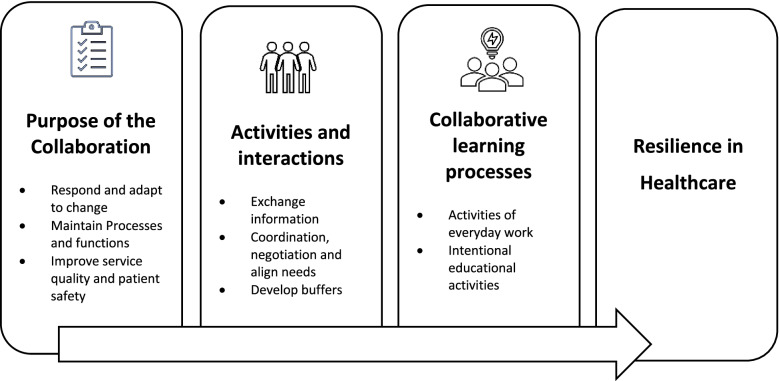
Collaborative processes, activities and learning processes in a resilient healthcare organization

In this paper we have presented and discussed the findings from a meta synthesis of collaborative processes and activities in healthcare and identified how these are aligned with learning in everyday practice as well as in response to changes at multiple system levels. As shown in Fig. 1, stakeholders in the healthcare system collaborate to respond and adapt to change, maintain processes and functions, and improve quality and safety. The activities and interactions that constitute these collaborations are exchange of information, coordination, negotiation, aligning needs, and developing buffers. The collaborative learning processes that arise from these activities and interactions are both activities of daily work, such as discussions, prioritizing and delegation of tasks, and intentional educational activities such as seminars or simulation activities. In the following section we discuss the findings as part of learning processes in resilient healthcare in light of resilient healthcare theory and learning theory.

### Collaborative working requires collaborative learning

Complex adaptive systems, such as healthcare, do not have the opportunity to fully plan every future event in advance. They depend on the ability of their healthcare professionals and teams to constantly adjust to emerging situations, creating safe processes continuously [1, 52–54]. Individuals in the system and their ability to anticipate, monitor, adapt and respond to potential threats are as such a valued asset to secure patient safety [48]. This ability to adapt is closely linked to the ability to learn, since adapting to a challenge or change is dependent on different kinds of contextual knowledge to handle the event [8, 55]. As shown in our findings, healthcare professionals often depend on collaborations within and across different system levels to adapt and respond. This is an understandable outcome of our healthcare systems being designed to provide healthcare as a collaborative effort [16]. The ability to adapt is, therefore, not just dependent on individuals’ actions, but also on collaborative efforts and the actions of the multiple stakeholders involved in the collaboration. Efforts to strengthen the ability to adapt should therefore be promoted and provided through a group context, where stakeholders who work together also learn together.

However, according to the definition of resilience, adopted here, the ability to maintain high quality care is dependent on the ability to adapt and respond at different system levels [7]. This means that efforts to strengthen resilience in healthcare need a systems perspective [7], and as such is not related to strengthening individual resilience. The fundamental issue for systems resilience, is how organizational processes can enable team or units to successfully collaborate to adapt to their changing circumstances [54]. Individual action is, therefore, an indirect reflection of systems resilience and is thus an important aspect that we need to understand. SH Berg and K Aase [56] and CJ Foster, KL Plant and NA Stanton [57] propose that resilient characteristics are interconnected both within and across different levels of a system. This interconnection implies that resilient capacities are dependent on a high level of collaboration within and across units, teams, contexts, and levels. Strengthening systems resilience, therefore, depends on continuous organizational based learning efforts that is inclusive of learning at both individual, team and organizational levels [58]. Improved system resilience through organizational learning is also dependent on the organizations ability to integrate changes more systematically into the everyday work of individuals but also groups and teams of stakeholders. The next level of organizational learning is, therefore, to create a shared understanding of how to address a challenge and why. Moreover, from a theoretical perspective, identifying mutual adjustments to diverse types of challenges emphasises the interactive and collaborative process needed to integrate the change into ordinary work practice [58]. Evidence of such mutual adjustment and shared understandings are identifiable in the findings of our synthesis through intentional learning or educational activities such as simulation and different forms of cross-level stakeholder meetings. However, our findings indicate that we need further investigations into how these learning processes at different levels are supported as part of enabling resilient performance.

From theoretical lenses, organizational based learning efforts, have often not occurred, until changes have been institutionalized and integrated in routines, rules and procedures [58]. As this study shows, such changes are dependent on collaborative processes between different stakeholders, who work and learn together, across different levels and contexts to maintain and improve healthcare provision. Efforts to strengthen organizational resilience should, therefore, focus on developing resilient capacities throughout all system levels and learning opportunities should be designed as collaborative efforts as this mimic their everyday collaborative work practice.

### Reflection and awareness—a key to successful adaptations

Findings from this study show that collaborative activities often consisted of different forms of trade-offs to get appropriate information, coordinate events, negotiate prioritizations, and align different needs. Similar to other resilience in healthcare studies [59, 60], this study found that the complex demands, competing interests and a diversity of stakeholders focusing on different outcomes resulted in the need to choose some type of adjustment of practice over another. While such trade-offs might be necessary to maintain situational processes and functions, adaptations and adjustments do not always provide positive outcomes for service quality and safety [2, 50]. In complex adaptive systems [4, 54], all individuals have a large degree of freedom to act in unpredictable ways. Furthermore, their actions interconnect, due to the high level of collaborative processes and as individuals’ actions influence each other’s, they also have consequences for other stakeholders in the system. So, what might appear as a rational action for individuals in a situation may have unforeseen consequences for others and move the system towards the boundaries of safe performance [1, 61]. Actions described in the findings, such as including next of kin in care responsibilities, consequently influenced the broader system by covering up a systemic error such as lack of staffing resources. This type of adaptation contributes to an increase in overall risk and limits the ability for resilient performance in a long-term perspective [50].

The complexity of the system, its everchanging circumstances and the interconnection through collaborative efforts, makes it difficult for individuals and teams to anticipate how to perform appropriate adaptations. However, individuals, teams, and organizations can be made aware of their role in such complex systems, and how local adaptations can have systemic consequences, and thereby aid decisions during negotiations and trade-offs through creating awareness of the impacts of specific choices. Enabling such learning processes could be a key to proactive approaches to quality and safety and the ability to monitor systems’ performance.

Recent research has indicated that the creation of reflective spaces, where different stakeholders have the opportunity for collaborative learning through meeting and exchanging experiences within and across levels has the potential to bridge tacit and explicit knowledge, and thereby create awareness [28]. Creating spaces for reflection that can facilitate mindfulness and awareness towards clinical practice, the choices that are made, and why, has been suggested as promising in other contexts [28, 38, 39, 62]. Creating reflexive spaces for stakeholders across different system contexts and levels could therefore potentially create higher awareness and understanding among different stakeholders related to how local adjustments could have systemic implications a in a complex adaptive healthcare system. Our findings support others that learning processes to a higher degree are embedded in the healthcare professionals every day work activities [25, 63]. Integrating reflexive spaces and awareness of what goes right in healthcare provision and why, as a part of healthcare professionals everyday work practice could potentially increase learning potentials within and across different levels in the healthcare system, and as a result contribute to strengthen resilient performance.

### Limitations

This meta-synthesis is based on a sample of 14 resilience narratives from a Norwegian setting. Including studies from only one country could have impacted the findings with local variations that are typical for the Norwegian context. While specifics of the Norwegian healthcare system in some respects differ from other international contexts, such as fewer private institutions and a government funded healthcare system, the collaborative learning processes which are studied in this paper, and how and why different actors in the healthcare system collaborate is believed to be representative of a broader healthcare context, and thereby also useful in an international context. Nevertheless, further studies are encouraged to be conducted with narratives based on a larger, more international sample of research studies. The choice of analysing narratives introduces the possibility that the interpreted material becomes misinterpreted or too distant from the intention of the original material. However, misinterpretation is always an issue in qualitative research and is therefore a potential bias that needs to be considered and counteracted throughout all qualitative research processes [64, 65]. This study has attempted to counteract this issue through a rigorous process both during the writing of the narratives and the analysis of the narratives, where a team of researchers with various backgrounds established a clear procedure on how to write the narratives and then continued to read, interpret and discuss to establish inter-rater reliability at all stages of the process [65]. The approach of meta-synthesis and combining methods for analysing data is also an important step that allows for multi-level and multi-setting research that can advance the field of resilience in healthcare [48, 66] Moreover, it allows for the inclusion of a large data set that provides the study with a rich data material in which to ground the results. However, future research should seek to include other studies from other setting to explore the role of collaborative learning in resilience in healthcare.

## Conclusion

The aim here was to describe collaborative learning processes in relation to resilient healthcare based on an investigation of narratives from diverse healthcare contexts and levels. The findings show that across levels and contexts healthcare workers collaborate to adapt and respond to changes, to maintain processes and functions, and to improve quality and safety. The activities and interactions these collaborations comprise are exchanging information, coordinating, negotiating, and aligning needs and developing buffers. All of which occur through collaborative working and are generative of learning and changes to practice. The learning activities embedded in these collaborations are both activities of daily work, such as discussions, prioritizing and delegation of tasks, in addition to intentional learning or educational activities such as seminars or simulations.

Based on our findings, we propose resilience in healthcare is dependent on these collaborations and learning processes, across different levels and contexts, to adapt and respond to challenges and changes and maintain high quality patient care. This ability to adapt is closely linked to the ability to learn. The resilience in healthcare approach holds a systems perspective. Although individuals’ actions are important, a systems perspective demands collaboration and learning within and across all system levels. Creating space for individual and collective appraisals and awareness building could assist individual, team, and organizational-based learning. Efforts to strengthen or further enable resilient performance should consider the importance of the collaborative element and seek to develop framework and learning tools that can facilitate learning through work and while working and learning together: that is collaboratively.

## Supplementary Information

**Additional file 1: Supplementary file 1. **Included projects. Overview of details of all the 14 projects included in the narratives, including their title, years of conduct, the setting in which empirical work occurred and informants.

## Data Availability

The datasets used and analysed during the current study are available from the corresponding author on reasonable request.

## Abbreviations

SHARE: Centre for Resilience in Healthcare
RiH: Resilience in healthcare
