# Supplementary material for: The role of collaborative learning in resilience in healthcare—a thematic qualitative meta-synthesis of resilience narratives
Source: BMC Health Serv Res. 2022 Aug 26;22:1091. doi: 10.1186/s12913-022-08451-y (PMC9412809; doi:10.1186/s12913-022-08451-y)
Supplement: Supplementary file 1 — Additional file 1: Supplementary file 1. Included projects. Overview of details of all the 14 projects included in the narratives, including their title, years of conduct, the setting in which empirical work occurred and informants. [file 12913_2022_8451_MOESM1_ESM.pdf]

Overview of included projects.

| Nr. | Project title | Year | Empirical setting | Involved Stakeholders |
|---|---|---|---|---|
| **1.** | QUASER Quality and Safety in European Hospitals | 2010-2013 | Hospitals | Healthcare personnel<br>Managers |
| **2.** | Next-of-kin involvement in regulatory investigation of adverse events that caused patient death | Finalized 2019 | Norwegian regulatory body<br>One county Governor's office in Norway | Next-of-kin<br>Regulatory investigators |
| **3.** | SAFE-LEAD | 2016-2021 | Nursing homes<br>Homecare services | Managers<br>Healthcare personnel |
| **4.** | Patient participation in transitional care of older patients | Finalized 2016 | Hospital admission, discharge, transitional care | Healthcare personnel (ambulance workers, nurses, and doctors)<br>Older patients |
| **5.** | Simulation-based telecare training for home healthcare professionals | Finalized 2019 | Homecare services | Homecare professionals |
| **6.** | Safe work practices in interdisciplinary surgical teamwork | Finalized 2013 | Hospital Surgical teamwork | Healthcare professionals |
| **7.** | Transitional care of the elderly from a resilience perspective | Finalized 2015 | Hospital, nursing homes, homecare services | Healthcare professionals, elderly patients |
| **8.** | Safe clinical practices for patients hospitalised in a suicidal crisis | Finalized 2020 | Psychiatric care in a Norwegian hospital | Suicidal patients<br>Healthcare professionals |
| **9.** | Next-of-kin involvement in hospital cancer care | Finalized 2021 | Cancer department in two hospitals. Managers and staff. | Next-of-kin, Healthcare professionals, Managers |
| **10.** | Exploring hospital readmissions from the primary healthcare service | Finalized 2020 | The interface between primary and secondary care. Hospitals, nursing homes. | Physicians, nurses and nursing home managers. |
| **11.** | Impact of active implementation of the Norwegian Musculoskeletal guideline on the use of non-traumatic musculoskeletal imaging | Finalized 2020 | Two municipalities in Norway. Hospital and general practitioners | GPs, radiologists, radiological fellows |
| **12.** | Safe use of telecare for older adults in homecare services | Ongoing | Six community homecare services in two Norwegian municipalities. | Homecare professionals (nurses and occupational therapists) |
| **13.** | Exploring links between resilience and macro-level development of healthcare regulation | Finalized 2021 | Employees at the ministry, the directorate, and the Norwegian board of health supervision | Actors at the macro level at Governmental regulatory bodies. |
| **14.** | A human factor approach to medication administration in nursing homes | Finalized 2020 | Nursing homes | Healthcare personnel in nursing homes involved in medication administration |
